# Cell–cell and cell–matrix dynamics in intraperitoneal cancer metastasis

**DOI:** 10.1007/s10555-012-9351-2

**Published:** 2012-04-13

**Authors:** Katharine L. Sodek, K. Joan Murphy, Theodore J. Brown, Maurice J. Ringuette

**Affiliations:** 1 Department of Cell and Systems Biology, University of Toronto, 25 Harbord St., Toronto, ON Canada M5S 3G5; 2 Division of Gynecologic Oncology, Princess Margaret Hospital, Toronto, ON Canada; 3 The Samuel Lunenfeld Research Institute, Mt. Sinai Hospital, Toronto, ON Canada; 4 Department of Obstetrics and Gynecology, University of Toronto, Toronto, ON Canada

**Keywords:** Peritoneum, Mesothelium, Ovarian cancer, Extracellular matrix, Collagen, Spheroid

## Abstract

The peritoneal metastatic route of cancer dissemination is shared by cancers of the ovary and gastrointestinal tract. Once initiated, peritoneal metastasis typically proceeds rapidly in a feed-forward manner. Several factors contribute to this efficient progression. In peritoneal metastasis, cancer cells exfoliate into the peritoneal fluid and spread locally, transported by peritoneal fluid. Inflammatory cytokines released by tumor and immune cells compromise the protective, anti-adhesive mesothelial cell layer that lines the peritoneal cavity, exposing the underlying extracellular matrix to which cancer cells readily attach. The peritoneum is further rendered receptive to metastatic implantation and growth by myofibroblastic cell behaviors also stimulated by inflammatory cytokines. Individual cancer cells suspended in peritoneal fluid can aggregate to form multicellular spheroids. This cellular arrangement imparts resistance to anoikis, apoptosis, and chemotherapeutics. Emerging evidence indicates that compact spheroid formation is preferentially accomplished by cancer cells with high invasive capacity and contractile behaviors. This review focuses on the pathological alterations to the peritoneum and the properties of cancer cells that in combination drive peritoneal metastasis.

## Introduction

Intraperitoneal dissemination is the primary metastatic route of ovarian cancers. It is also a common progression for gastrointestinal malignancies including colorectal, gastric, and pancreatic cancers, for which it signifies a grim prognosis [1, 2]. The poor prognosis relates, in large part, to the rapid progression of peritoneal metastasis in comparison to the hematological (blood-borne) metastatic route. This latter route, recently reviewed [3, 4], is a more laborious process that involves cancer cell penetration of multiple barriers during intravasation and extravasation from blood vessels, as well as growth in a foreign environment. The distinct mechanisms involved in peritoneal metastasis contribute to its devastating efficiency, and are the focus of this review.

While all cells within the ovary can give rise to malignancies, epithelial ovarian cancers (EOC) are the most common and lethal. EOCs are a heterogeneous group of cancers that may be categorized into two major groups [5]. Type I EOCs are low-grade, slow-growing tumors that are thought to arise from benign ovarian lesions and include all four major histotypes: serous, endometrioid, mucinous, and clear cell. While the cell of origin for these cancers remains controversial, a favored model is that they develop from the ovarian epithelium. When segments of the ovarian surface epithelium or other Mullerian-derived epithelia become entrapped within cortical inclusion cysts in the ovary, they are exposed to a hormone-rich environment that promotes the tumorigenesis of cells possessing oncogenic mutations (e.g., KRAS, BRAF, β-catenin, or TGFβRII). In contrast, type II EOCs may be derived from the secretory cells of the fallopian tube epithelia and have tubal rather than ovarian precursor lesions. Type II EOCs frequently have p53 mutations and are, hence, genetically unstable and present histologically as high-grade serous, mixed epithelial, or undifferentiated carcinomas. These cancers are thought to seed the ovarian surface and pelvic peritoneum concurrently, which explains why they rarely present as stage I disease [5].

EOCs generally have an insidious onset. Due to the asymptomatic nature of early-stage disease, most patients are not diagnosed until after their tumors have metastasized intraperitoneally. At this point, their chance of surviving beyond 5 years is only about 25 % [6], which is largely due to the diffuse peritoneal lesions that impede surgical eradication. In fact, the completeness of surgical debulking is the best predictor of survival. Chemotherapy, while initially effective, ultimately fails to prevent disease progression because patients almost inevitably develop recurrent resistant disease [7, 8].

Intraperitoneal metastases can cause peritoneal organ adhesion and malfunction, massive ascites, and/or pleural effusions [9–11], leading to mortality. In contrast, the hematological metastatic route is not a significant contributor to EOC mortality [12], reflecting the relative ease and speed of cancer dissemination and growth in the peritoneal cavity. Colorectal and gastric cancers can metastasize through hematological, lymphatic, or intraperitoneal routes. These epithelial cancers typically arise on the luminal/mucosal side of the gastrointestinal tract, which is well separated from the serosal (peritoneal) membrane. However, cancer cells can shed directly into the peritoneal cavity for tumors that breach the submucosa, smooth muscle, and serosal layers. Alternatively, accidental perforation of the intestinal wall during surgery can introduce cancer cells from tumors that were previously not exposed to the peritoneal space. Irrespective of the means of introduction, peritoneal involvement drastically worsens the prognosis for these patients [13, 14].

A key factor contributing to the poor prognosis of intraperitoneally metastasizing cancers is the rapid, self-perpetuating, feed-forward cycle of seeding and growth that is fuelled by inflammation. Since the prognosis of patients with peritoneal metastases is tightly correlated with the completeness of surgical cytoreduction [15, 16], and widespread metastases are not amenable to surgery, the development of novel strategies to arrest metastatic progression is imperative. This review summarizes the current understanding of mechanisms involved in peritoneal metastasis, with a focus on altered cell–cell and cell–matrix dynamics.

## The peritoneum is receptive to metastatic growth

The mechanisms involved in peritoneal metastasis are distinct from those of the hematological route. Relative to hematological metastasis, peritoneal metastasis is a passive, efficient process that occurs locally in a self-perpetuating, feed-forward cycle. This presents unique challenges to intervention.

The peritoneal cavity is particularly receptive to metastasis, as evidenced by several key observations. First, although EOC cells are detected in the circulation at relatively early stages of disease [12], metastatic deposits outside of the peritoneal cavity are rare and not a common cause of morbidity. Overwhelming evidence for the tumor-receptive environment can be gleaned from cases where pressure from massive ascites was relieved using a perito-venous shunt. This procedure introduced vast numbers of malignant cells into the circulation, yet it did not place patients at increased risk of mortality. Postmortem autopsies revealed either a lack of extraperitoneal metastasis, or when present, lesions that were small and clinically asymptomatic. This surprising finding indicates that cancer cells capable of metastasis in the abdominal cavity are incapable of significant growth in other tissues, supporting the notion that the peritoneal environment is receptive to cancer seeding [17].

Additional evidence that the peritoneal environment provides a privileged site for cancer metastasis is reflected in prognostic data. Colorectal carcinoma prognosis is dramatically worsened by accidental bowel perforation during surgery [18, 19], a mishap that can directly introduce cancer cells into the peritoneal cavity. In gastrointestinal cancers including gastric, appendiceal, colonic, and rectal, the involvement of the serosal membrane (which is synonymous to, and contiguous with, the peritoneal surface) is one of the most important determinants of overall prognosis [14]. In gastric cancer, the presence of tumor cells in peritoneal lavages is a predictor of decreased survival time, whereas micrometastases in lymph nodes or bone marrow are of limited prognostic value [20]. For endometrial cancer, there are recent cautions that the practice of diagnostic hysteroscopy, which involves increasing intrauterine pressure using distension media, may retrogradely drive cancer cells into the peritoneal cavity through the fallopian tubes, thereby increasing the risk of metastasis [21]. Collectively, these observations strongly support the peritoneum as a particularly receptive environment for cancer metastasis and growth.

## Intraperitoneal dissemination of cancer cells

Cancer cells can freely disseminate in the peritoneal cavity after exfoliating from exposed primary intraperitoneal tumors: ovarian epithelia for type I EOC, fallopian tube epithelia for type II EOC, and the serosal membrane for colorectal or gastric cancers. They also gain access when introduced through accidental surgical perforation of the bowel wall [22]. In either case, once suspended in the peritoneal fluid, the cancer cells must resist anoikis, a specialized form of apoptosis triggered by a lack of attachment to other cells or to the extracellular matrix (ECM). They must also evade clearance through the peritoneal lymphatics. By attaching to the peritoneal membrane, cancer cells avoid both anoikis and clearance. Accordingly, interactions between tumor cells and the peritoneum are key contributors to metastatic progression, which, if successfully blocked, should promote the clearance or death of cancer cells.

## Cellular and molecular properties of the peritoneum

The peritoneum is comprised of a single layer of mesothelial cells and its associated underlying ECM, which cover the vast surface of the abdominal and pelvic cavities, as well as visceral organs (∼1.7 m^2^, comparable to the surface area of the skin). Mesothelial cells apically secrete glycosaminoglycans (primarily hyaluronan), surfactant (mainly phosphatidylcholine) and proteoglycans to provide an anti-adhesive peritoneal surface that ensures the appropriate gliding of the abdominal viscera and prevents intra-abdominal organ fusion [23]. Intra-abdominal adhesions are a complication that frequently occurs with peritoneal surgery due to the unavoidable disruption of this fragile mesothelial layer [24]. Consistent with their anti-adhesive functioning, mesothelial cells also protect against cancer cell attachment, which will be further discussed.

Mesothelial cells regulate the entry of leukocytes and inflammatory cells into the peritoneal cavity. In response to injury or insult, these cells release chemokines MCP-1 and IL-8 and upregulate cell surface adhesion molecules ICAM-1 and VCAM-1, to which leukocytes attach [25].

Mesothelial cells possess both epithelial and mesenchymal characteristics and readily undergo epithelial–mesenchymal transformation (EMT) and myofibroblast transformation in response to normal tissue repair and pathological stimuli [23]. The ECM underlying the peritoneal mesothelial cells is rich in collagen I and fibronectin, with thin deposits of laminin and collagen IV lying directly beneath the mesothelium [26]. The ECM is, for the most part, concealed by the flattened squamous-like mesothelial cell layer; however, it is periodically exposed at the lymphatic portals through which the peritoneal fluid drains into the venous circulation. These lymphatic portals are particularly abundant on the omental and sub-diaphragmatic peritoneal surfaces and are commonly referred to as “milky spots” because of their whitish appearance that results from the accumulation of resident lymphocytes participating in immune surveillance [25]. Ultrastructural analysis reveals the absence of a basement membrane at milky spots. Instead, the collagen I-rich stromal matrix is exposed. The adjacent mesothelial cells have a cuboidal morphology with disruptions and intercellular gaps that further expose the peritoneal ECM. This cuboidal morphology likely reflects activation by cytokine secretions from the neighboring milky spot lymphocytes [23, 27].

## Cancer cell attachment to the peritoneum

Metastasizing cancer cells have two main options for attachment to the peritoneum: the surface mesothelial cells or the exposed ECM. Many investigations have focused on the mechanisms mediating cancer cell attachment to a cultured mesothelial monolayer. Some studies suggest that the mechanisms normally used for leukocyte attachment to mesothelial cells may be exploited by cancer cells. Ovarian, colorectal, and pancreatic cancer cells bind to mesothelial cell surface receptors ICAM-1 and/or VCAM-1 [28–30], which are upregulated in response to injury or insult [25]. As well, the hyaluronan receptor CD44, expressed by many cancer cell types including ovarian and gastric cancer cells, enables these cells to bind to the hyaluronan-rich apical surface of mesothelial cells [31–34]. The ovarian cancer biomarker CA125/MUC16 is a transmembrane mucin that binds to mesothelin, a GPI-linked protein expressed by mesothelial cells [35]. Hence, a variety of different adhesion molecules can mediate the attachment of cancer cells to mesothelial monolayers. Attachment of cancer cells to the ECM, on the other hand, is mainly mediated by integrins. The β1 integrin subunit is key as it can pair with a variety of α-integrin subunits to confer binding to most ECM substrata. Blocking β1 integrin inhibits EOC cell attachment and migration on ECM substrata relevant to the peritoneum [36, 37]. β1 integrins also participate in cancer cell attachment to mesothelial monolayers [33, 37–39], which could reflect that cancer cells are binding to the mesothelium-associated ECM and/or to mesothelial cell surface VCAM-1, as outlined above. Cancer cell lines display differential reliance on specific adhesion molecules. Therefore, the specific cell lines and *in vitro* model system selected have greatly influenced study conclusions.

Ensuring that the *in vitro* models are an accurate representation of the *in vivo* events is crucial for identifying meaningful targets for intervention. Many studies have been designed with the assumption that peritoneal metastasis relies on cancer cell attachment to mesothelial cells. However, other studies indicate that cancer cells have a much greater affinity for the peritoneal ECM, which is consistent with the clinical pattern of metastatic spread.

## Mesothelial cells protect against cancer cell attachment

**Fig. 1:**
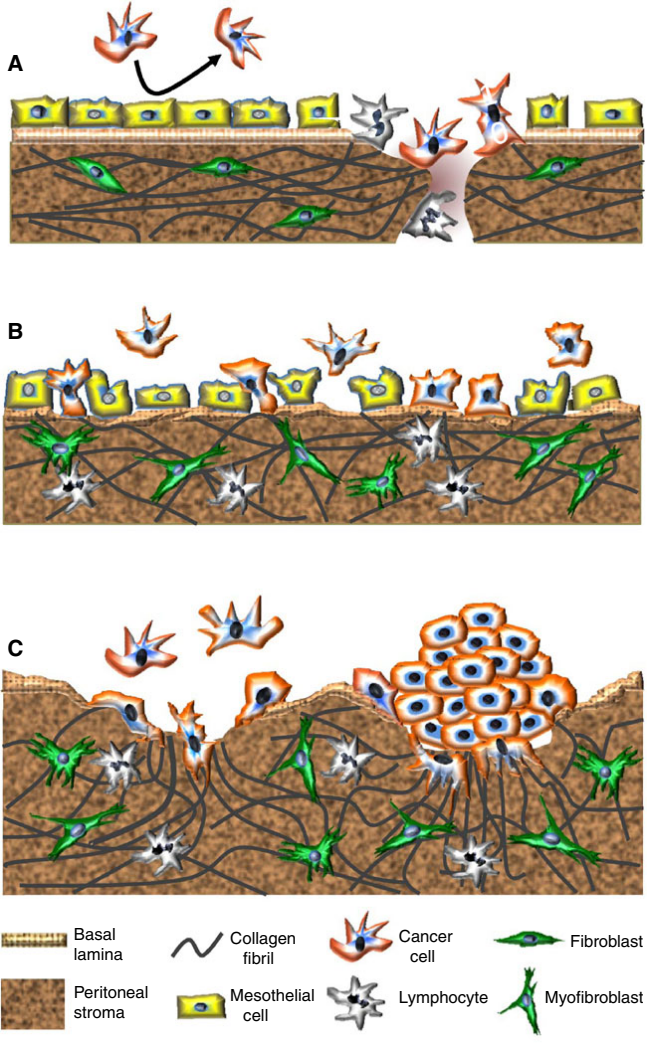
Changing patterns of metastatic spread with disease progression. **a** Cancer cells initially attach to milky spots where the stromal matrix is exposed, providing direct access to their preferred substrate, collagen I. The intact mesothelial layer discourages cancer cell attachment. **b** With disease progression and in response to increasing concentrations of inflammatory mediators, mesothelial cells retract and detach. The resulting exposure of the underlying ECM, with a discontinuous basement membrane, facilitates widespread peritoneal metastasis. TGF-β, released by cancer and inflammatory cells, stimulates myofibroblast transdifferentiation. **c** Metastasizing cancer cells, particularly those with a highly invasive, contractile phenotype, form compact spheroids in peritoneal fluid. This protects them against anoikis and chemotherapeutics. These spheroids attach to and invade the peritoneal matrix. The combination of their contractile and proteolytic capacities remodels the collagen I-rich matrix to facilitate stromal implantation and invasive growth

Several lines of evidence indicate that mesothelial cells protect against, rather than mediate, cancer cell attachment. Collagen I, a major constituent of the sub-mesothelial ECM [26], is the preferred substrate for ovarian cancer cell attachment [40] and migration [37, 41]. Moreover, collagen I binding activates EOC cell invasive behavior [42]. EOC cell lines with an aggressive phenotype have an elevated expression of the α2 and β1 collagen-binding integrin subunits as compared to cells with lower invasive capacity [43]. For gastric cancer cells, peritoneal invasion was inhibited by blocking the collagen I-binding integrin α2β1 [44]. Hence, the mesothelial layer actively discourages cancer cell attachment by occluding the underlying collagen I-rich extracellular matrix to which cancer cells preferentially attach (Fig. 1).

Surgical trauma and stress damage the mesothelium and expose the underlying ECM, creating privileged sites for cancer cell attachment [45, 46]. The benefit of peritoneal lavage following surgery to wash out exfoliated tumor cells may be countered by the damage to the fragile mesothelial cell layer that enhances metastasic spread [47]. The enhanced peritoneal invasion that occurs in response to surgical trauma is mediated by β1 integrins [48]. In support of the concept that mesothelial cells discourage rather than mediate cancer cell attachment, Kenny et al. [49] determined that cancer cell adhesion to a 3D reconstituted omental stromal matrix was inhibited when an overlying layer of mesothelial cells was included in the model. Conversely, cancer cell adhesion to *ex vivo* omental tissue was markedly elevated when the mesothelial layer was removed.

The clinical pattern of disease progression underscores the concept that cancer cells preferentially attach to areas where the mesothelium is disrupted. During the initial stages of peritoneal metastasis, cancer cells attach to milky spots where the collagen-rich connective tissue matrix is exposed [50–53] (Fig. 1a). The resident immune cells of the milky spots are not able to prevent tumor growth [50, 54]; instead, their production of pro-inflammatory cytokines promotes cancer growth and dissemination. The abundance of milky spots within the omentum might explain the predilection of cancer cells to seed this structure. The omentum also contains a large number of adipocytes that may promote the growth of the attaching cancer cells by providing lipids to meet their energy demands [55].

## Inflammatory alterations render the peritoneum susceptible to tumor cell adhesion

Secretions from cancer, stromal, mesothelial, and immune cells, particularly macrophages, contribute to an inflammatory environment that drives peritoneal metastasis [56–58]. Consistent with the action of soluble inflammatory cytokines and chemokines, widespread alterations in gene expression reflecting a more adhesive peritoneum were found in peritoneal tissue from patients with EOC [59, 60].

The impact of inflammatory cytokines on peritoneal metastasis is profound and transforms the initial pattern of dissemination, which is limited to milky spots, into a widespread peritoneal metastasis [51, 61]. This transformation is triggered by an increased exposure of the sub-mesothelial ECM, driven by inflammation. The inflammatory cytokines, tumor necrosis factor alpha (TNFα) and interleukin 1β (IL1β) cause the protective mesothelial cells to retract, exposing the previously obscured underlying ECM. Since cancer cells preferentially attach to the ECM, widespread cancer cell attachment ensues [51, 61] (Fig. 1b).

TNFα is highly expressed by cancer and inflammatory cells [51, 62, 63], and its levels are elevated in peritoneal effusions of ovarian cancer patients [64]. The associated retraction and uplifting of mesothelial cells is characteristic of EOC patients [65]. In addition to causing mesothelial retraction, TNFα and other inflammatory mediators upregulate ICAM-1 and VCAM-1 adhesion molecules on mesothelial cells, which facilitates cancer–mesothelial cell interactions [30]. Despite this, when cancer cells were added to a monolayer of mesothelial cells that had been pretreated with TNFα, the cancer cells attached to the intracellular gaps where the sub-mesothelial collagen I matrix was exposed rather than to the mesothelial cells [51]. This finding underscores the preference of the cancer cells to attach to the collagen-rich ECM.

In light of the preference of cancer cells for the sub-mesothelial matrix, it is unclear why blocking interactions between cancer cells and mesothelial cells impedes metastasis in experimental models. Antibody-mediated inhibition of CD44 [34, 66] for ovarian and colorectal cell lines expressing high levels of CD44, or of VCAM [30], slowed the progression of peritoneal metastasis in mice. In these experiments, the blocking agents were administered simultaneously with a large bolus of cancer cells to mice that presumably had a healthy, intact peritoneal mesothelial layer. This is in marked contrast to the situation in most patients who present with advanced disease and peritoneal inflammatory alterations.

Collectively, the evidence supports an inhibitory role of the mesothelium in peritoneal cancer cell attachment (Fig. 1). While activated mesothelial cells may be less efficient at creating a barrier than a quiescent mesothelial monolayer, in either case, these cells protect against cancer cell adhesion by concealing the underlying connective tissue matrix to which cancer cells preferentially attach. Knowing that cancer cells prefer areas where the mesothelium is absent and the peritoneal ECM is exposed highlights the importance of targeting cancer cell interactions with the ECM while simultaneously preventing mesothelial cell retraction.

## Ascites formation contributes to peritoneal metastasis

Ascites, an accumulation of protein-rich exudate within the abdominal cavity, is a complication that often accompanies cancers metastasizing within the peritoneum. In EOC, ascites is frequently a presenting feature of advanced-stage disease, and although it often resolves following chemotherapy, it generally re-accumulates in chemo-resistant and recurrent disease. In other cancers, ascites is a late event when the goal of treatment is palliation [67]. Ascites facilitates a widespread dissemination of cancer cells in the abdominal cavity [68] and has been correlated with a poor prognosis. In a study limited to patients with stage III/IV EOC, women without ascites had a 5-year survival rate of 45 % compared to 5 % for those with ascites [69].

Clinically, ascites is a distressing and debilitating complication that significantly impacts quality of life [70]. The volume of ascites varies widely among patients, ranging from <100 mL to in excess of 10 L quantities that can cause considerable discomfort and contribute to organ dysfunction. In cases where massive re-accumulation of ascites occurs, as is common with chemo-resistant EOC, repeated paracentesis for temporary palliation is required. Removal of large volumes of ascitic fluid carries with it the risk of hypovolemic shock and contributes to the development of inanition, while repeated paracentesis increases the risk of bowel perforation and peritonitis. Diuretics may be administered, and, in rare cases, peritoneo-venous shunts or indwelling drainage catheters are surgically installed to enable drainage. Unfortunately, there is no established gold standard for the clinical management of malignant ascites [71, 72].

The pathophysiology of ascites accumulation involves increased net filtration and/or decreased drainage of peritoneal fluid; that is, fluid production is more rapid than its clearance [73]. Ascites thus can arise through multiple processes, including the blockage of lymphatic channels draining the peritoneum by metastatic cells, increased permeability of capillaries within the peritoneal wall due in large part to the actions of VEGF, decreased protein levels in the blood resulting in increased fluid movement to the abdominal cavity, and hepatic portal vein compression or liver failure due to massive liver metastasis. Notably, it is the tumor-free peritoneal surface that provides the majority of the surplus fluid in cancer-associated ascites [74].

A positive feedback cycle of cytokine release that involves cancer, mesothelial, and inflammatory cells contributes to ascites [75]. In EOC, cancer cells secrete VEGF-A and monocyte chemoattractant protein-1, which attract monocytes that contribute more VEGF-A (plus other cytokines and growth factors). VEGF-A increases vascular permeability and neovascularization. A large body of evidence implicates VEGF in ascites formation [75–78] (reviewed in [79]). Accordingly, aflibercept, a soluble circulating VEGF receptor decoy that acts as a VEGF trap, holds promise for the relief of malignant ascites based on the outcomes of two recent clinical trials [80, 81], the latter of which was a randomized, double-blind placebo-controlled study.

**Fig. 2:**
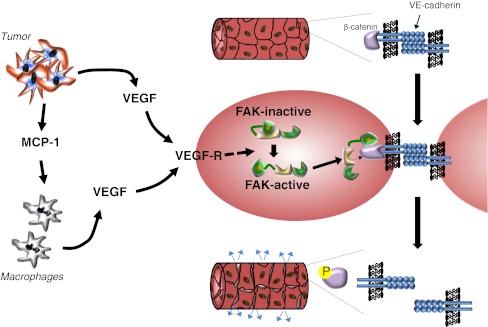
VEGF-induced vascular permeability leads to the accumulation of ascites. Tumor cells secrete VEGF and chemokines such as MCP-1 that attract macrophages, which secrete additional VEGF. VEGF binds to its receptor on vascular endothelial cells in the peritoneal wall, leading to the activation of focal adhesion kinase (*FAK*). Activated FAK binds to the tail of the VE-cadherin that mediates endothelial cell–cell junctions and phosphorylates β-catenin, triggering its release which destabilizes these junctions [82]. The resulting increase in vascular permeability is a major contributor to ascites formation and accumulation

A cellular mechanism by which VEGF increases vascular permeability has recently been revealed [82]. VEGF binding to its receptor activates focal adhesion kinase (FAK) which localizes to the cytoplasmic tail of VE-cadherin at endothelial cell–cell junctions. FAK phosphorylates β-catenin, which destabilizes the cell–cell junctions, resulting in increased vascular permeability (Fig. 2).

Cancer-associated ascites is enriched in other growth factors and pro-inflammatory factors secreted from cancer, reactive stromal, and inflammatory cells that contribute to the aforementioned peritoneal inflammatory alterations that promote cancer cell attachment. These include IL-8 [61, 83], IL-6 [84], TNFα [64], TGFβ, IL-12, and IL-10 [76], hepatocyte growth factor (HGF) [85], heparin-binding epidermal growth factor (HB-EGF) [86], and lysophosphatidic acid (LPA) [87]. Several of these factors as well as others found in ascites may promote chemoresistance [83, 88, 89]. Interestingly, ascites from different patients was found to have variable effects on the motility of ovarian cancer cells, with a subset of these samples containing a heat-sensitive inhibitory substance [90]. Fibrinogen and fibrin also enter the peritoneal cavity in increased amounts during ascites formation [91]. In contrast to the beneficial effect of fibrin deposition in immune defense, where it serves to help entrap intraperitoneal bacteria [76], fibrin deposition is detrimental in the case of peritoneal cancer because it entraps metastasizing tumor cells, leading to the formation of aggregates that become vascularized metastatic deposits in a process facilitated by the elevated VEGF-A and IL-8 [91].

## Proteolysis in peritoneal metastasis

Peritoneal metastasis, in contrast to hematologic metastasis, does not require cancer cells to degrade matrix barriers associated with intravasation and extravasation of the vasculature. Rather, peritoneal metastasis entails cell adhesion, highly variable degrees of invasive growth and/or carpet-like spreading on peritoneal surfaces. The degree of invasion into the stroma varies and may be related to EOC grade and histotype, although the determinants of invasive behavior are undoubtedly multifactorial. Irrespective of invasive depth, proteolysis is required for cancer cells to anchor and establish metastatic implants.

Cancer cell interactions with basal lamina components are probably not a prime determinant of peritoneal metastasis since the cancer cells have direct access to their preferred substrate, the fibrillar collagen I-rich peritoneal stroma, at milky spots and in areas exposed by mesothelial cell retraction. The collagenolytic matrix metalloproteinase (MMP) subset, which includes MMP-1, MMP-8, MMP-13, and the MT-MMPs, is therefore implicated in invasion. While the importance of MMPs in EOC metastasis is recognized [92, 93], clinical trials using the broad-range MMP inhibitors marimastat and tanomastat have yielded disappointing results [94–96]. This likely relates to the fact that the inhibitor concentrations attained were far below levels required to accomplish the inhibition of critical MMPs [97, 98]. Moreover, certain MMPs can, depending on the microenvironmental context, counter tumor progression such that their inhibition would be counterproductive [99]. Thus, a more targeted approach that accomplishes the inhibition of specific cancer-promoting MMPs should be of therapeutic advantage [100].

A deterministic role for the transmembrane subgroup of MMPs (MT-MMPs) has been revealed for cancer cell transmigration through stromal and basement membrane matrices, which pose protease-dependent barriers to cell migration *in vivo* (reviewed by [101]). MT-MMPs acting at the leading edge of invading cells effect a focal pericellular proteolysis of the matrix barriers while leaving the matrix sufficiently intact to support cell migration. Soluble MMPs do not cleave fibrillar collagens in a manner that is conducive to cancer cell invasion, but may facilitate this process by cleaving cell surface receptors or by generating bioactive products in the surrounding stroma [101].

MT1-MMP (MMP-14) has been revealed as a critical determinant of EOC cell invasion through collagen I matrices [102] where cell capacities for invasion reflected those reported *in vivo* in an intraperitoneal xenograph murine model [103]. MT1-MMP was also revealed to be an important mediator of peritoneal metastasis by gastric cancer cells, and the authors noted that metastasis by cancer cells lacking MT1-MMP was rare [104].

Studies using collagen I [87, 90] and extensive studies by Weiss and colleagues using *ex vivo* peritoneal membrane and mammary gland explants [82, 83] exclude the need for soluble MMPs in matrix invasion by cancer cells. However, MMP-2 was reported to enhance the early adhesion of ovarian cancer cells to the peritoneum by cleaving fibronectin and vitronectin to create a superior substratum for α5β1 and αvβ3 integrin binding [105]. As MT1-MMP is the key activator of pro-MMP-2 [106], this implies an additional mechanism for its contribution to peritoneal metastasis.

In addition to degrading the ECM and activating soluble MMPs, MT1-MMP promotes peritoneal metastasis through its sheddase activity. One such target is the transmembrane epidermal growth factor HB-EGF. This growth factor has a prominent role in peritoneal metastasis, and its elevated expression is correlated with a poor clinical outcome [107]. HB-EGF overexpression confers intraperitoneal metastatic ability to cancer cells that were otherwise incapable of metastasis. Conversely, its inhibition greatly reduces intraperitoneal metastatic progression [108]. CRM197, a mutated diphtheria toxin that specifically binds and inactivates HB-EGF, and inhibits peritoneal metastasis in mice, is now in phase I clinical trial [109]. HB-EGF is shed by ADAMs (a disintegrin and metalloproteinase) to release an active soluble growth factor that is fully active only when bound to heparan sulfate proteoglycans. This soluble fragment can be further cleaved by MT1-MMP to generate a dually processed potent form that does not require heparan cofactors for activity [110]. This potent form of HB-EGF is present in EOC patient ascites [111]. The invasive growth of gastric and ovarian cancer cells, as well as their anchorage-independent growth depends on MT1-MMP-mediated HB-EGF cleavage [110, 111]. Efforts to develop small-molecule inhibitors of MT1-MMP have been stimulated by compelling evidence that this protease drives the progression of multiple cancer types [112]. As these specific inhibitors become available, it will be possible to better assess the role MT1-MMP in peritoneal metastasis.

## Cell contractility promotes metastasis

Cells maintain a reciprocal dialogue with their surrounding ECM through integrin receptors which indirectly link the ECM to the actin cytoskeleton. Integrin activation can be mediated by growth factor “inside-out” activation or ECM-mediated “outside-in” activation. The clustering of activated integrins triggers signaling cascades involving FAK, Src, PI3K, RhoA, Rho kinase (ROCK), and myosin regulatory light chain (MLCK) phosphorylation, culminating in actinomyosin-mediated cell contractility, which transmits forces that alter the ECM. Tissue homeostasis is dependent on a balance between ECM deposition and turnover. When this delicate balance is disrupted by growth factors and cytokines associated with wounding or cancer, a feed-forward cycle of increased ECM deposition, rigidity, and cell contractile behavior ensue, which perpetuates peritoneal metastasis in several ways, as discussed below.

The dense, rigid matrices formed through excessive collagen deposition and actomyosin-mediated contraction strongly promote cell proliferation and tumorigenesis [113–117]. Rigid matrices facilitate cancer metastasis by supporting scattering behavior. The stronger “foothold” they provide enables integrin-associated actomyosin forces to overcome and sever cell–cell attachments, a prerequisite to the emigration and invasion of individual cells [117, 118]. Cell-mediated contraction also contributes to metastasis in a 3D environment by re-orientating collagen fibers into “highways” that are used as tracks for cancer cell migration and invasion [119, 120]. In support of this concept, invasive regions of tumor explants remodeled their surrounding ECM to achieve a radial orientation of collagen fibrils, upon which they migrated [121]. Similarly, ovarian cancer cell lines with robust contractile behavior were the only ones capable of migrating through 3D collagen I matrices [122].

Contractile behavior by surrounding stromal cells can also facilitate metastasis. Force-mediated matrix remodeling by fibroblasts was required for squamous cell carcinoma cell invasion through a mixed collagen I-based matrix [123]. Moreover, ovarian cancer cell invasion though a reconstituted omentum was enhanced when fibroblasts were included in the collagen I matrix, an effect that could not be recapitulated by substituting fibroblast-conditioned media. While the authors speculated that cell–cell contact between cancer and fibroblast cells was involved in the enhanced migration [49], an alternative possibility is that matrix contraction by the fibroblasts, perhaps in response to stimulation by the cancer cells, contributed to a reorganization of the matrix that promoted cancer cell invasion. The cells best known for matrix deposition and contractility, myofibroblasts, are associated with many tumor types, including ovarian [124, 125], and are known to drive cancer progression [126, 127].

## Derivation of tumor-associated myofibroblasts

Tumor-associated myofibroblasts originate from several precursor cell types, including fibroblasts, mesothelial cells, and cancer cells, and contribute to ECM alterations that promote metastasis. Fibroblast–myofibroblast transdifferentiation is a well-recognized phenomenon that contributes to numerous pathologies [126, 127]. In the case of cancer, factors secreted by cancer cells initiate the myofibroblast transdifferentiation through a process that involves chloride intracellular channel 4 (CLIC4) upregulation as a prerequisite to the expression of the myofibroblast marker alpha smooth muscle actin (αSMA) [128]. TGF-β is a key activator of the transdifferentiation of fibroblasts into myofibroblasts. This cytokine is secreted by many cancer cell types, including ovarian, and contributes to the upregulation of αSMA [125, 129].

The transdifferentiation of mesothelial cells into myofibroblasts is also stimulated by TGF-β. This process is commonly observed in peritoneal dialysis patients and leads to loss of the mesothelium, fibrosis, and peritoneal membrane failure [130, 131]. A similar transdifferentiation could explain the loss of mesothelial and fibroblast cells and the gain of αSMA-positive myofibroblasts at sites of invasive tumor implantation in the peritoneum [132]. Bone marrow-derived circulating cells and endothelial cells are also activated by TGF-β and contribute to the peritoneal myofibroblast population [131]. Moreover, there is compelling evidence that epithelial cancer cells transdifferentiate into myofibroblast-like cells in an EMT-dependent process [133].

## Contractile behavior enhances cancer cell survival by promoting compact spheroid formation

Contractile behavior may be particularly beneficial for the survival of cancer cells during peritoneal metastasis. When suspended individually during transit in the peritoneal fluid, cancer cells are susceptible to anoikis and other apoptotic triggers. However, by combining with other suspended cells to form compact multicellular “spheroid” aggregates, these cells can resist anoikis and apoptosis, including that induced by chemotherapeutics. The abundance of integrin attachments available to cells within the 3D spheroid configuration is thought to be a major contributor to pro-survival signaling [134, 135].

Spheroids, which range from 50 to 750 μm in size [38], are commonly found in the peritoneal fluid or ascites of EOC patients, independent of histotype [136]. As a source of viable metastatic cells, spheroids readily adhere to and disaggregate on ECM substrates, particularly collagen I, as well as on mesothelial cell monolayers [36, 37]. A growing recognition of the importance of spheroids in EOC dissemination has stimulated increased interest in their formation and function. An intriguing positive association between contractile behavior, compact spheroid-forming ability, and the invasive capacity of cancer cells in 3D compliant matrices has been revealed [122].

## Compact spheroid formation is preferentially accomplished by invasive cancer cells

Cancer cells possess varying capacities for spheroid formation [38, 122, 137]. Whereas a positive correlation between spheroid formation and tumorigenicity has been suggested [137], an inverse association between spheroid cohesiveness and invasive potential has been reported for brain tumor cells [138]. The possibility that cells in spheroids gain invasive properties by undergoing EMT while in a spheroid arrangement has been proposed [139]. However, compact spheroid formation was found to be accomplished only by EOC cell lines that already possessed a mesenchymal phenotype. These cell lines also had a superior migratory and invasive capacity compared to the cell lines that did not form compact spheroids, properties that existed prior to, as well as subsequent to, spheroid formation [122]. These results suggest that an aggressive cancer cell subpopulation is able to gain a survival advantage through its propensity for compact spheroid formation. Any further enhancement of invasive potential gained within the spheroid microenvironment, as proposed [139], would add to this detrimental scenario. A likely basis for the relationship between compact spheroid formation and cell migration/invasion within 3D compliant matrices is that both processes rely on contractile cell behavior [122].

## Mechanisms involved in spheroid formation

As a first step in spheroid formation, cells must interact with one another either directly or through ECM bridges. An ECM network interconnects EOC cells within spheroids [38], and integrin attachments to fibronectin, collagen, and Matrigel support spheroid formation by different cancer cell types [38, 140–143]. Direct cell–cell attachments through homotypic cadherin associations can also mediate spheroid formation [140, 144]. Hence, a variety of cell adhesion mechanisms can mediate the initial aggregation of cancer cells into spheroids.

The compaction of aggregated cells into dense spheroids is dependent upon the contractile capacity of the cells [122]. A similar phenomenon, the compaction of spheroid-like microtissues and toroid formations [145], is mediated by actinomyosin forces. Both integrins and cadherins have been implicated in spheroid formation, and as each provides links to the actin cytoskeleton to transmit actomyosin-mediated forces, it is likely that actomyosin-mediated contraction is universally involved in spheroid compaction.

## Malignant ascites promotes compact spheroid formation

**Fig. 3:**
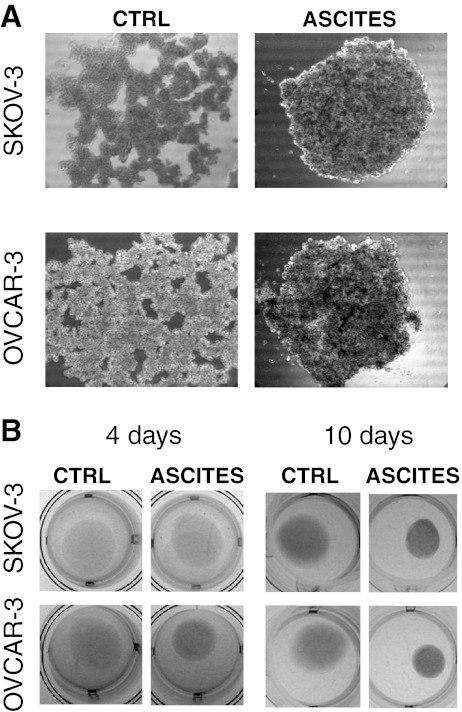
Ascites promotes compact spheroid formation and contractile behavior by human ovarian cancer cell lines otherwise incapable of these behaviors. **a** Enhanced spheroid formation by human ovarian cancer cell lines, SKOV-3 and OVCAR-3, in response to treatment with malignant ascites from a patient diagnosed with advanced epithelial ovarian cancer. Spheroids were formed by the hanging drop method and culture medium (containing 10 % serum) was supplemented with ascites (1:1, *v*/*v*). Images were obtained after 3 days. **b** Enhanced collagen gel contraction by SKOV-3 and OVCAR-3 cells in response to treatment with malignant ascites. Cells were mixed with collagen type I (Vitrogen) for a final concentration of 5 × 10^5^ cells/1.6 mg Vitrogen per milliliter. Results are shown at 4 days (*left panel*) and 10 days (*right panel*). While ascites had a pronounced effect on these parameters, these changes were modest compared to the compact spheroid formation and collagen gel contraction exhibited by the more invasive cell lines in the absence of ascites [122]

ECM molecules, growth factors, and cytokines present in ascites, including fibronectin, TGF-β, HGF, EGF, and LPA, promote cell adhesion and motility [56, 146] and induce contractile behavior by fibroblasts [118, 147–149]. EOC cells that otherwise do not form compact spheroids are able to do so when the tissue culture media (containing 10 % serum) is supplemented with ascites from a patient with high-grade serous EOC (Fig. 3a). Since ascites supplementation also enabled their collagen gel contraction (Fig. 3b), it is likely that the stimulation of cell contractility by ascites was an important contributor to the enhanced spheroid formation. Notably, the collagen gel contraction and spheroid formation promoted by ascites in the EOC cell lines normally incapable of these behaviors was modest compared to the abilities possessed by contractile, invasive EOC cell lines [108], even in the absence of ascites exposure. Thus, while ascites components can augment these behaviors, the genetic program of the cancer cells themselves remains a prime determinant of contractile and spheroid forming capacity.

## Cell contractile behavior provides protection against chemotherapeutics

In solid tumors, contractile behavior creates dense rigid matrices that confer drug resistance by (1) increasing the potential for integrin attachments and their associated pro-survival signaling [116] and (2) generating a high interstitial hydrostatic pressure that hinders drug delivery [150]. Contractile behavior would also endow chemoresistance to intraperitoneally disseminating cancer cells by promoting their incorporation into compact spheroids. Cells in spheroids are known to have an enhanced resistance to many antitumor chemotherapeutics. This phenomenon of “multicellular resistance” has been attributed to multiple mechanisms, including a decreased penetrance of therapeutics, decreased cell proliferation, increased pro-survival integrin signaling, and an upregulation of genes conferring drug resistance [134, 135, 151, 152].

Strong support for a role of compact spheroid formation in conferring drug resistance is provided by a study that compared drug-resistant cells to their parental cell lines. Cells selected for drug resistance *in vivo* showed a striking ability to form compact spheroids as compared to their parental cells. Moreover, their enhanced resistance to chemotherapeutics was evident *in vitro* only when they were compared to the parental cells as a spheroid culture. When cultured as monolayers, differences in drug resistance were admonished [153]. This provides compelling evidence that the chemoprotective effect was conferred by compact spheroid formation. Coupled with the evidence that aggressive cancer cells have an increased propensity for compact spheroid formation [122], and given the challenge of chemoresistant disease in EOC [154], these studies underscore the importance of preventing compact spheroid formation as a means of diminishing peritoneal metastasis and enhancing the efficacy of current drug therapies. Since cells can use a variety of potentially redundant mechanisms for their initial aggregation into spheroids (e.g., integrin–ECM or cadherin-mediated) [140], targeting this phase is likely to require a multifaceted approach. In contrast, actomyosin contractility, a likely common denominator and critical mediator of spheroid compaction, is attractive as a potential therapeutic target.

## Targeting actomyosin contractility in peritoneal metastasis

Cell contractile behavior potentially drives peritoneal cancer progression through several mechanisms. By inhibiting actomyosin contractility, the increases in matrix density and rigidity that promote malignant progression and interfere with the delivery of chemotherapeutics could be reduced. Moreover, cells in transit should be sensitized to anoikis and chemotherapeutics if compact spheroid formation could be prevented.

ROCK plays a major role in cell contractile behavior. ROCK ensures myosin II remains active and capable of binding and contracting actin filaments by (1) phosphorylating and inactivating myosin regulatory light chain phosphatase and (2) promoting myosin regulatory light chain phosphorylation directly, as well as indirectly by phosphorylating MLCK. ROCK also activates LIM kinase, which phosphorylates and inactivates cofilins, thus preventing F-actin severing [155]. Two isoforms of ROCK have been identified, ROCK1 and ROCK2. These isoforms share high sequence identity in their kinase domain [156].

The conditional overexpression of ROCK2 in murine skin increased tissue stiffness and thickening and enhanced tumor incidence, growth, and progression. These effects were reversible when ROCK2 or its downstream effectors (myosin ATPase, LIM kinase) or its upstream activator FAK was inhibited [117]. The cell behaviors mediated by ROCK, including cell motility, adhesion, and contraction, have been implicated in a range of diseases, and the clinical benefit of ROCK inhibition with Fasudil (HA-1077), which targets both ROCK isoforms, is under investigation [155].

ROCK1/2 inhibition using Y-27632 interfered with a process similar to spheroid formation: the cell sorting and compaction involved in microtissue formation [145]. Y-27632 protects against TGF-β-induced mesothelial EMT [157] as well as glucose-induced peritoneal fibrosis [158] in experiments using a rodent model. This suggests that, at least for the peritoneal membrane and its stromal counterparts, ROCK inhibition could be beneficial for reducing the inflammatory changes associated with cancer, including mesothelial retraction.

While it is tempting to speculate that ROCK inhibition could slow peritoneal metastatic progression by blocking spheroid compaction and motile behavior of cancer cells, the observation that ROCK inhibition enabled embryonic stem cells [159] and prostate stem cells [160] to resist anoikis is noteworthy. Establishing the precise outcome of ROCK inhibition on cancer cell behaviors, under both attached and anchorage-independent/spheroid culture conditions, is therefore crucial.

## Future perspectives

High-throughput genetic and proteomic approaches hold promise for the discovery of early-stage disease biomarkers that may improve survival rates. However, identifying disease-specific markers capable of heralding early events in carcinogenesis has been proven to be a formidable challenge. Effective therapies to combat metastatic disease are urgently required and will continue to be needed, independent of gains in our ability for early detection, as screening approaches cannot be expected to detect all cancers at early stages. While many ovarian cancers are initially responsive to current chemotherapeutic approaches, progression to a drug-resistant form is an almost universal occurrence. Novel strategies that target cancer cell interactions with the peritoneal ECM and inflammation-driven peritoneal modifications that expose the peritoneal ECM are likely to be broadly applicable to cancers that metastasize within the abdominal cavity.

A cornerstone to devising strategies to block peritoneal metastasis is the selection and implementation of suitable *in vitro* and *in vivo* invasion models that accurately mirror the clinical situation. Cancer cells preferentially attach to and invade regions of the peritoneum where the sub-mesothelial ECM is exposed rather than to an intact mesothelium. This is evident in the pattern of peritoneal metastasis. Initially, cancer cells disseminate to milky spots where the basement membrane is disrupted and where they have direct access to the collagen I-rich connective tissue matrix. Collagen I is arguably the most important ECM component with which cancer cells interact during peritoneal dissemination, both for adhesion and invasion.

Mounting evidence indicates that cell behavior in 3D culture differs from monolayer culture and better reflects the *in vivo* situation [161]. Consideration must be given to the choice of ECM used in these cultures. Many commercially available collagen I solutions fail to provide a physiologically relevant barrier to cell migration when reconstituted as 3D gels. For ease of extraction, collagen I is often proteolytically digested to remove the telopeptide regions that contain the majority of intra- and intermolecular collagen I cross-links. As a result, cells can migrate through this reconstituted matrix in the absence of proteolytic activity since the matrix comprises a loose collection of collagen fibrils [98, 162–164]. Similarly, Matrigel, a laminin and collagen IV-rich extract from Engelbreth–Holm–Swarm tumor cells that is often used as a surrogate basement membrane, is unsuitable for studying cell invasion. Cell penetration of Matrigel, unlike that of bona fide basement membranes, occurs in the absence of MMP activity [162, 164, 165]. Basement membrane assembly is a cell-mediated process that uses precise and extensive covalent cross-linking and disulfide bond formation to form a stable sheet-like network of collagen IV. While Matrigel is rich in laminin and collagen IV, it lacks the stable laminar organization that is a hallmark of basement membrane matrices [101, 162]. Hence, penetration through Matrigel assesses cell migration rather than cell invasion.

Collagen I with intact telopeptide regions can be isolated by acid extraction and reconstituted as a 3D gel that forms a true (MMP-dependent) barrier to cell transmigration. A limitation is that the density and rigidity found *in vivo* are not reflected *in vitro* due to the limited solubility of collagen, which is an important consideration in light of compelling evidence that matrix density and rigidity have fundamental effects on cell behavior and differentiation [113, 166, 167]. *Ex vivo* peritoneal matrices retain a requirement for MMP-driven invasion as well as a density and complexity that resembles the *in vivo* situation [97]. It would be informative to explore differences in the mechanisms of cancer cell attachment to membranes obtained from animals with and without pre-established peritoneal inflammation.

Strategies that block tumor cell interaction with the sub-mesothelial ECM (rather than the mesothelium itself) should prove more effective in blocking intraperitoneal metastatic dissemination at clinically relevant disease stages since cancer cells preferentially adhere to exposed peritoneal ECM. In addition, minimizing the amount of exposed peritoneal ECM by preventing mesothelial retraction is an intriguing avenue to further reduce peritoneal metastasis [168, 169]. If cancer cell–ECM interactions could be limited by minimizing mesothelial cell retraction, the approach of blocking mesothelial–cancer cell interactions would become more relevant.

Cells that gain a survival advantage by forming compact spheroids likely represent an aggressive subpopulation of cells, at least for EOC. This underscores the importance of targeting spheroid formation. Whether invasive capacity and compact spheroid formation are linked in other intraperitoneal cancers should be determined. The likelihood that compact spheroid formation and cancer cell-induced ECM remodeling both involve cellular contractility presents the exciting possibility that chemoresistance and invasion can be simultaneously targeted.

## Summary

Several events conspire to promote peritoneal metastasis:

1. The peritoneal cavity is highly susceptible to tumor cell implantation and growth. Cells need only to exfoliate from primary tumors and secondary sites into the peritoneal fluid, which provides efficient transport to additional sites within the abdominal cavity. The presence of milky spots/lymphatic ducts, where a basement membrane is lacking and the adhesive collagen-I-rich connective tissue matrix is directly exposed, encourages the attachment of metastasizing cells.
2. Cancer cells stimulate a pro-inflammatory response within the peritoneal cavity, which augments metastasis. Inflammatory mediators cause retraction and uplifting of the protective mesothelial cell lining, which promotes cancer cell attachment by exposing the underlying pro-adhesive ECM. Inflammatory mediators also cause myofibroblast transdifferentiation of mesothelial cells and fibroblasts and initiate a fibrotic response within the peritoneum. The dense, rigid, fibrotic matrices that result promote tumor cell survival, growth, invasion, and chemoresistance by enhancing integrin signaling.
3. MT1-MMP has emerged as an important contributor to peritoneal metastasis. In addition to its established pericellular collagenolytic activity that is crucial for invasive growth, its conversion of HB-EGF into a more potent, heparin-independent form, as well as its activation of soluble pro-MMP-2, likely contribute to its ability to promote intraperitoneal metastasis.
4. Invasive cancer cells likely attain resistance to anoikis and chemotherapeutics by virtue of their propensity for contractile behavior and compact spheroid formation. This link between cancer cell invasive ability and capacity for compact spheroid formation highlights the importance of targeting these multicellular aggregates.
